# Broesike hernia: long-standing incharacteristic abdominal pain

**DOI:** 10.1590/0100-3984.2017.0001

**Published:** 2018

**Authors:** Alinne Christina Alves Pires, Diogo Costa Oliveira, Marcelo Souto Nacif, Marcelo Fontalvo Martin, João Maurício Canavezi Indiani

**Affiliations:** 1 Unidade de Radiologia Clínica (URC), São José dos Campos, SP, Brazil.; 2 Universidade Federal Fluminense (UFF), Niterói, RJ, Brazil.

*Dear Editor,*

**Figure 1:**
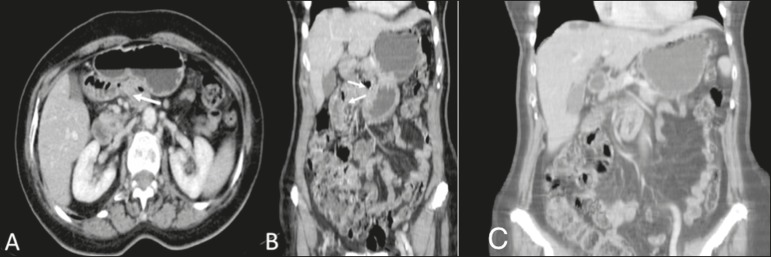
Contrast-enhanced portal-phase MDCT of the abdomen, in the axial plane (A) and coronal plane (B). Note the normally positioned third portion of the duodenal arch and the fourth portion passing through the right mesocolic fossa (arrow to the right), representing a mobile ligament of Treitz, and with jejunal loops predominantly on the right side. C: MIP reconstruction in the coronal plane, showing fewer small bowel loops to the left of the mesenteric vessels.

A 75-year-old woman presented with a three-year history of nonspecific, moderate, intermittent mesogastric pain, similar to colic, with little improvement after medication, accompanied by nausea and postprandial fullness. She had a history of abdominal surgery, with a resurgence of symptoms six months prior to seeking treatment at our facility, and the attending physician therefore requested a multidetector computed tomography (MDCT) scan (Figure 1) for diagnostic purposes. On MDCT, the first, second, and third portions of the duodenal arch were seen to be in their normal positions, whereas the fourth portion passed through the right mesocolic fossa (mobile ligament of Treitz), with jejunal loops predominantly on that side, crossing over to the contralateral side to the mesenteric vein and artery. That finding is characteristic of right paraduodenal hernia through the mesocolic fossa (Broesike hernia), with no signs of obstruction of the jejunal loop in the MDCT examination, with high suspicion due to the intermittent aspect of this finding.

**Figure 2:**
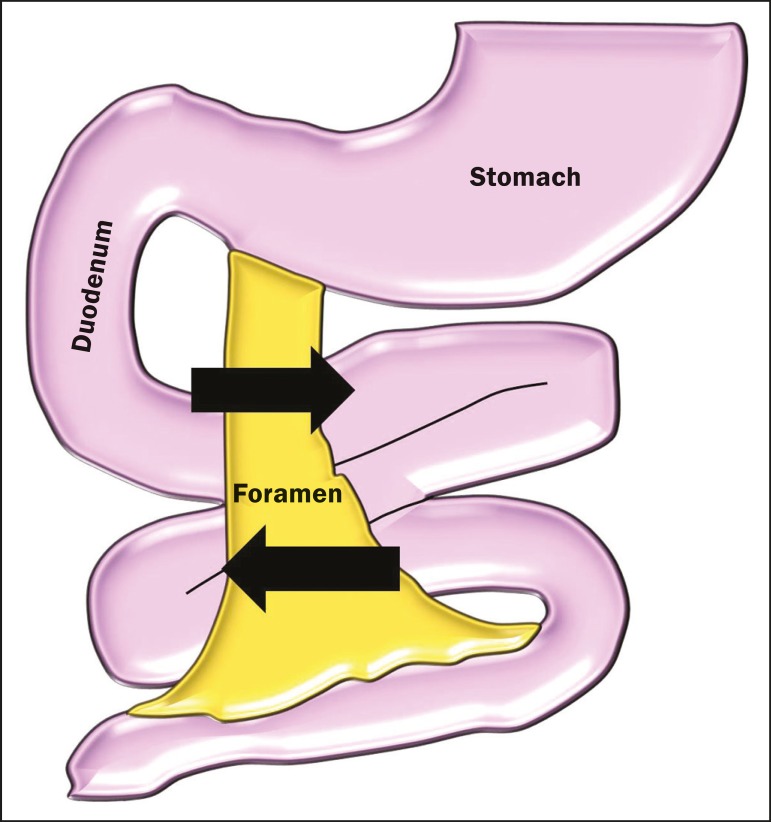
Schematic of the mechanism of the occurrence of right paraduodenal hernia. The proximal portion of the small bowel remains to the right, insinuating itself into the hernia recess, known as the Waldeyer fossa, posterior and inferior to the opening. That mobility can promote slippage of the loops, and in more severe cases, their strangulation.

The greater anatomical characterization capacity of MDCT and the development of diagnostic imaging methods have made the study of internal abdominal hernias more accurate, given that intermittent symptoms could exclude their diagnosis^(1-3)^. Caused by congenital mechanisms, surgery, trauma, inflammation or poor circulation, they have several subclassifications^(4,5)^. Right paraduodenal (or Broesike) hernia is defined as one in which the small bowel is concentrated in the peritoneal cavity, adjacent to the ligament of Treitz^(2,4)^. They develop through the Waldeyer fossa, resulting from fusion failure of the ascending mesocolon with posterior parietal peritoneum^(3)^ (Figure 2).

Despite affecting approximately 50% of patients diagnosed with internal hernia, paraduodenal hernias tend to affect elderly males in larger proportions, although Broesike hernias account for 25% of such cases, most frequently being attributable to malrotation of the small bowel^(1-3,5)^. Certainly underdiagnosed, Broesike hernia is a rare cause of intestinal obstruction and is difficult to diagnose from a clinical, surgical, and radiological perspective; a delay in its diagnosis leads to catastrophic outcomes, such as acute obstruction of the small bowel, ischemia, and intestinal perforation^(1,2,6)^. It can be asymptomatic or provoke symptoms ranging from vague constant epigastric pain to intermittent colic-like periumbilical pain (accompanied by nausea and recurrent intestinal obstruction) to incarceration or strangulation. Its typically intermittent symptoms make the medical team question the veracity of patient complaints^(5)^.

When identified by MDCT, hernia with intestinal subocclusion shows an “encapsulated” mass of dilated small bowel between the pancreas and stomach, to the right of the ligament of Treitz^(2,5)^. In general, the mass dislocates the posterior wall of the stomach, the duodenal flexure, and (inferiorly) the transverse colon. There is engorgement of the mesenteric vessels, which supply segments of the small bowel in the area of the hernia defect^(3,5)^. In the case described here, MDCT revealed laterally insinuated loops clustered in the region of the mesocolic fossa, with no significant distension or injury of the jejunal loops. The addition of contrast could increase the diagnostic accuracy of the radiological examinations.

Laparoscopic repair is a safe and effective method for the management of cases of Broesike hernia^(7,8)^. However, because the patient described here was at prohibitive surgical risk, we opted for clinical monitoring. At this writing, the patient has been followed at our facility for over six months, without any worsening of her symptoms.
